# Cellular Basis of Pineal Gland Development: Emerging Role of Microglia as Phenotype Regulator

**DOI:** 10.1371/journal.pone.0167063

**Published:** 2016-11-18

**Authors:** María P. Ibañez Rodriguez, Stephen C. Noctor, Estela M. Muñoz

**Affiliations:** 1 Institute of Histology and Embryology of Mendoza (IHEM), National University of Cuyo, National Scientific and Technical Research Council (CONICET), Mendoza, Argentina; 2 Department of Psychiatry and Behavioral Sciences, MIND Institute, University of California, Davis, School of Medicine, Sacramento, CA, United States of America; Universite Claude Bernard Lyon 1, FRANCE

## Abstract

The adult pineal gland is composed of pinealocytes, astrocytes, microglia, and other interstitial cells that have been described in detail. However, factors that contribute to pineal development have not been fully elucidated, nor have pineal cell lineages been well characterized. We applied systematic double, triple and quadruple labeling of cell-specific markers on prenatal, postnatal and mature rat pineal gland tissue combined with confocal microscopy to provide a comprehensive view of the cellular dynamics and cell lineages that contribute to pineal gland development. The pineal gland begins as an evagination of neuroepithelium in the roof of the third ventricle. The pineal primordium initially consists of radially aligned Pax6+ precursor cells that express vimentin and divide at the ventricular lumen. After the tubular neuroepithelium fuses, the distribution of Pax6+ cells transitions to include rosette-like structures and later, dispersed cells. In the developing gland all dividing cells express Pax6, indicating that Pax6+ precursor cells generate pinealocytes and some interstitial cells. The density of Pax6+ cells decreases across pineal development as a result of cellular differentiation and microglial phagocytosis, but Pax6+ cells remain in the adult gland as a distinct population. Microglial colonization begins after pineal recess formation. Microglial phagocytosis of Pax6+ cells is not common at early stages but increases as microglia colonize the gland. In the postnatal gland microglia affiliate with Tuj1+ nerve fibers, IB4+ blood vessels, and Pax6+ cells. We demonstrate that microglia engulf Pax6+ cells, nerve fibers, and blood vessel-related elements, but not pinealocytes. We conclude that microglia play a role in pineal gland formation and homeostasis by regulating the precursor cell population, remodeling blood vessels and pruning sympathetic nerve fibers.

## Introduction

In vertebrates, the pineal gland effects and regulates the circadian timing system by transducing environmental light into an internal signal, the nocturnal melatonin [1]. The pineal gland develops from a committed area of the neuroepithelium that lines the roof of the third ventricle in the prenatal brain, and its maturation continues postnatally. During the first postnatal week the rat pineal gland begins responding in a rhythmic fashion to sympathetic innervation from the superior cervical ganglia [2,3], which relay circadian information from the suprachiasmatic nuclei (SCN). While a growing body of work has identified cellular and transcriptional mechanisms required for pineal ontogeny, additional factors that contribute to pineal development and homeostasis have not been fully elucidated.

A dynamic and intricate regulatory network of transcription factors drives the definition and maintenance of pineal phenotype [4–10]. The homeobox transcription factors Pax6, Otx2 and Lhx9 are necessary for proper pineal gland formation [11–16]. Pax6 is considered one of the earliest phenotype determinants responsible for regulating pinealocyte specification and prenatal proliferation since the pineal gland fails to develop in the absence of functional Pax6. Rath et al. demonstrated that Pax6 mRNA expression peaks in the developing rat pineal gland on embryonic (E) day 18, followed by a rapid perinatal decline [17]. However, the cells that express the Pax6 protein have not been fully characterized in terms of their location, distribution, function or relationship with other cells in the pineal gland. In addition, the Pax6+ cell lineage fate throughout pineal development has not been well delineated. In this study we present the ontogeny of the Pax6+ cell lineage in the rat pineal gland, and how interactions between Pax6+ cells and other pineal gland cell types contribute to gland formation and homeostasis.

The mature pineal gland is considered a relatively homogeneous organ that is composed of a small set of well-defined cell types. Approximately 95% of the cells are pinealocytes, with the remainder consisting mainly of interstitial cells–astrocytes and microglia–embedded in a network of blood vessels and nerve fibers [18]. The concept of pinealocyte homogeneity, however, is currently being reevaluated [10,19]. Microglia have been identified as one of the pineal interstitial cell types via OX6 (MHCII), OX42 (CD11b), IL-1β, ED1 (CD68), and TNF-R1 expression, among other markers [20–26]. Microglia have been reported to play several roles in the pineal gland, including regulation of pinealocyte neurites in a cytokine-dependent manner [27–30]; serving as antigen-presenting cells [20,22]; sensing physical injury, bacteria, and hypoxia [21,31,32], and modulating pineal melatonin [31–35]. Our data expand the repertoire of microglial functions in the developing and adult pineal gland. We show that microglia phagocytose Pax6+ cells, especially in the adult gland, and also engulf blood vessel and nerve fiber elements. Our data provide a novel perspective on the cellular dynamics that shape formation of the developing pineal gland and homeostasis in the mature pineal gland.

## Materials and Methods

### Animals

All animal procedures performed in this study were in agreement with the National Institutes of Health’s Guide for Care and Use of Laboratory Animals, the Animal Research: Reporting in Vivo Experiments (ARRIVE) Guidelines, and with approval by the Institutional Animal Care and Use Committee at the School of Medicine, National University of Cuyo, Mendoza, Argentina (Protocol ID 9/2012). All efforts were made to minimize the number of animals used and their suffering. Embryonic, neonatal, juvenile and adult male Wistar rats were processed. In the prenatal period female embryos from timed pregnant mothers were also included. Animals were housed in temperature and humidity-controlled rooms under a 12:12 light:dark (L:D) cycle and free access to food and water. Rats were euthanized according to age either by decapitation after hypothermia by immersion in wet ice [36] or ketamine/xylazine (50 and 5 mg/kg of body weight, respectively) anesthesia. Samples at embryonic day (E) 15, 16, 17, 18, 19, 20, 21, and postnatal day (P) 3, 7, 9 and 90, were collected during the light phase at *Zeitgeber* time (ZT) 6 and immediately processed for immunohistochemistry [10,37].

### Immunohistochemistry

Samples for immunostaining were fixed in 4% paraformaldehyde (PFA) in phosphate-buffered saline (PBS) at 4°C. Entire E15 embryos, whole E16 heads, and adult pineal glands were fixed by immersion. Complete brains, including pineal glands, from exsanguinated late embryos and neonatal rats were dissected after transcardial perfusion with the same fixative mixture. After fixation, the organs were rinsed twice in PBS, dehydrated in increasing concentrations of ethanol (from 50% to 100%), washed twice in xylene, immersed in a mixture of 1:1 xylene and Histoplast (Biopack, Bs. As., Argentina), and finally embedded in Histoplast. The incubation time in each solution varied according to the tissue size. Ten-micrometer sections from fixed samples were cut using a Microm HM 325 microtome (Thermo Fisher Scientific Inc., Waltham, MA, USA). Slide-mounted tissues were hydrated in decreasing concentrations of ethanol (from 100% to 50%) and pure distilled water, and then subjected to antigen retrieval by boiling in 0.01 mM sodium citrate buffer (pH6) containing (v/v) 0.05% Tween-20 for thirty minutes. Non-specific labeling was avoided by using blocking solution [(v/v) 10% donkey serum, 1% Triton X-100 and (w/v) 0.2% gelatin in PBS] for 1 hour at room temperature (RT) in a humid chamber. Immunodetection was performed using primary antibody buffer [(v/v) 2% donkey serum, 1% Triton X-100, and (w/v) 0.2% gelatin in PBS] containing primary antibodies (see below), overnight at RT. Primary antibodies were as follows: mouse monoclonal anti-ED1 1:100 (MCA341GA, AbD Serotec, Bio-Rad Laboratories Inc., Hercules, CA, USA); goat polyclonal anti-Iba1 1:200 (ab5076, Abcam, Cambridge, MA, USA); rabbit polyclonal anti-Pax6 1:200 (Previously Covance PRB-278P, BioLegend, San Diego, CA, USA); mouse monoclonal anti-PCNA 1:50 (MAB424, EMD Millipore, Bilerica, MA, USA); mouse monoclonal anti-phosphoSer^10^-histone H3 (PH3) 1:100 (ab14955, Abcam); goat polyclonal anti-serotonin 1:500 (ab66047, Abcam); mouse monoclonal anti-neuronal class III β–tubulin (Tuj1) 1:500 (Previously Covance MMS-435P, BioLegend), and mouse monoclonal anti-vimentin (VIM) 1:200 (V6630, Sigma, St. Louis, MO, USA). Isolectin GS-IB4 (IB4) from *Griffonia simplicifolia* conjugated with Alexa Fluor 568 1:100 (I21412, Life Technologies-Invitrogen, CABA, Bs. As., Argentina) was also included in this study. After incubation with primary antibodies, sections were rinsed three times for five minutes each in PBS, and then incubated in secondary antibody buffer with the nuclear dye DAPI 1:400 (D1306, Life Technologies-Invitrogen) for 2 hours at RT. Secondary antibodies with low cross-reaction generated in donkey and conjugated with Alexa Fluor 488, Cy3/Dylight 549 and Alexa Fluor 647, were used at a dilution 1:200 (Jackson InmunoResearch Laboratories Inc., West Grove, PA, USA). Slices were rinsed and then covered with Mowiol. Primary antibodies were routinely omitted to determine non-specific labeling. The optimal antiserum concentrations were defined by initially immunostaining tissue sections with each primary antibody alone using the dilution series recommended by the manufacturers. We then performed our double, triple and quadruple stainings with the cocktails of primary antibodies, comparing results with those obtained from single antibody immunostainings. Imaging was performed on an Olympus FV1000 confocal microscope (Olympus America Inc., Center Valley, PA, USA). Images were processed with MacBiophotonics ImageJ and edited with Adobe Photoshop 7.0 (Adobe Systems Inc., San Jose, CA, USA). Analysis of Z-stack images through the ten micrometer thickness confirmed phagocytosis of Pax6+ cells, Tuj1-immunoreactive nerve fibers, and IB4-labeled blood vessel elements. In the figures, phagocytosis events are shown in single confocal planes, projections from sequential confocal planes and 3D reconstructions.

### Analysis of Pax6-immunoreactive precursors and Iba1-positive microglial cells

Pineal gland sections from E20, P3, P9 and adult (P90) rats were immunolabeled for Pax6 and Iba1. Images were captured with the Olympus FV1000 confocal microscope using a 40x objective and processed with the MacBiophotonics ImageJ software. The proportions of total Pax6+ precursor cells, total Iba1-immunolabeled microglia, and precursor cells in close proximity or in the process of being phagocytosed by microglia, were quantified in an area of 0.05 mm^2^. The counted squares with at least one microglia body were randomly distributed in the selected images. Five pineal glands per developmental stage and a number of images per pineal gland that varied with age (from two at E20 up to six in adult pineal glands) were processed. Data were expressed as mean ± S.E.M. The statistical analysis included one-way ANOVA followed by the test for linear trend and the Tukey post-test, and was performed using PRISM5 (GraphPad Software Inc. La Jolla, CA, USA). *P* < 0.05 was considered significant.

## Results

**Fig 1:**
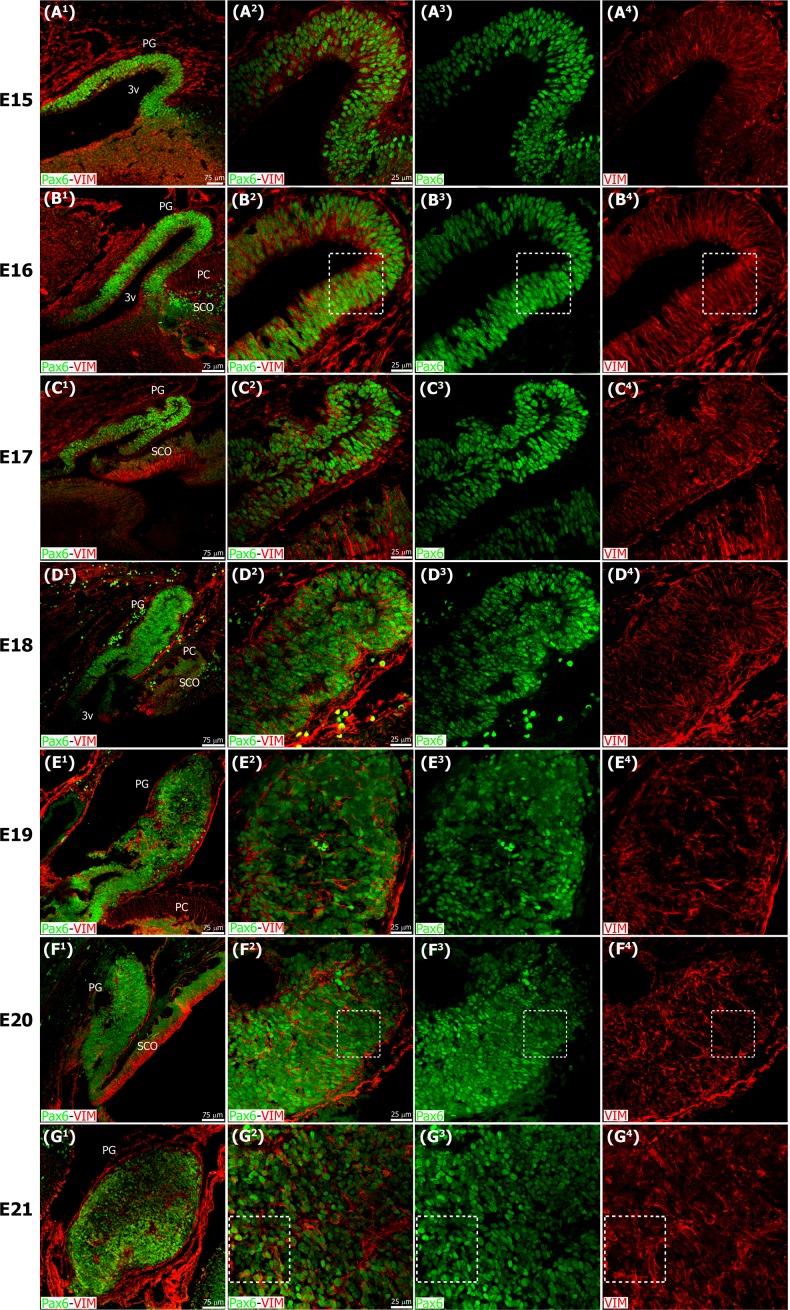
The pineal gland develops from neuroepithelial cells that express the transcription factor Pax6 and the intermediate filament vimentin. Panels display confocal microscopy of immunolabeled sagittal sections of rat pineal gland (PG) from embryonic day (E) 15 to E21. Both male and female rat embryos were used. Panels A^1^-G^1^ show expression of Pax6 (green) and vimentin (VIM, red) in pineal precursor cells at low magnification. Panels A^2^-G^2^ display the same structures at higher magnification. Panels A^3^-G^3^ and A^4^-G^4^ show Pax6 and vimentin expression for each stage of development, respectively. Pineal organogenesis begins around E15 as an evagination of the neuroepithelium in the dorsal diencephalon that is densely populated by Pax6-expressing cells (green). The developing PG becomes a tubular extension at E16. The orientation of Pax6/VIM+ cells is radial at these stages. At E17 the pineal neuroepithelium begins to fold and fuses at the midline. After fusion of the neuroepithelium, double immunolabeled rosette-like structures are visible in the E18-E21 developing PG. At E21 the PG has developed into a recognizable globular structure. Inset boxes are shown at higher magnification in Fig 3. (A^1^-G^1^) 20x; scale bar: 75 μm. (A^2^-G^4^) 60x; scale bar: 25 μm. PC, posterior commissure. SCO, subcommissural organ. 3v, third ventricle.

**Fig 2:**
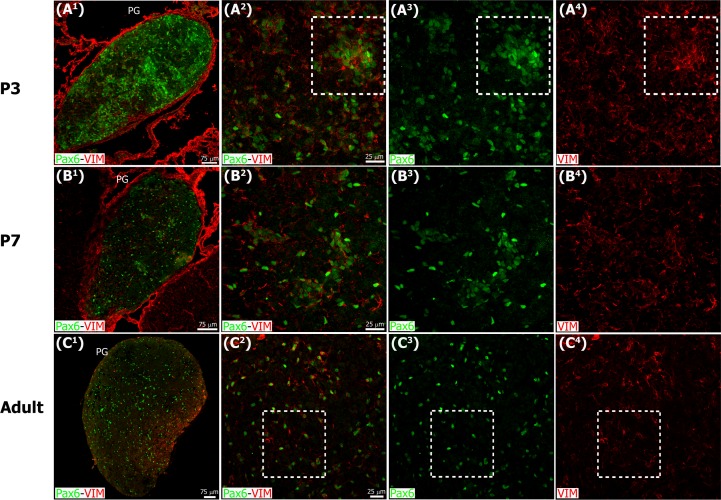
Pax6 and vimentin expression persists in the postnatal and adult pineal gland. Immunoreactivity for Pax6 (green) and vimentin (VIM, red) in pineal glands (PG) from 3-, 7- and 90-day-old male rats (P3, P7 and adult, respectively). The levels of expression of both markers appear to decrease throughout PG development. Pax6+ and/or VIM+ cells are still organized in discrete rosette-like patches at P3, while dispersed immunoreactive cells are more abundant from P7 onwards. Inset boxes are shown at higher magnification in Fig 3. (A^1^-B^1^) 20x; (C^1^) 1.2x digital zoom from a 10x image; scale bar: 75 μm. (A^2^-B^4^) 60x; (C^2^-C^4^) 40x; scale bar: 25 μm.

**Fig 3:**
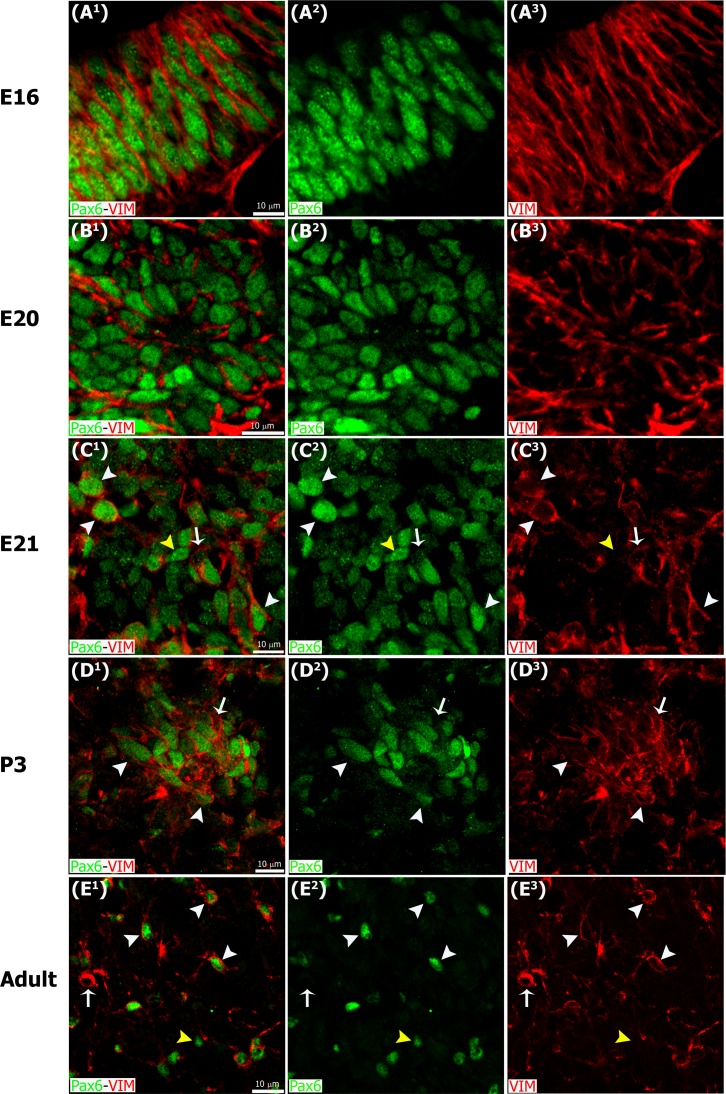
Pax6/Vimentin double immunostaining reveals dynamic changes in the organization of precursor cells throughout pineal ontogeny. Panels include higher magnification images of the insets shown at 60x and 40x in Figs 1 and 2 immunostained for Pax6 (green) and vimentin (VIM, red). (A^1^-A^3^) In the earliest stages Pax6/VIM double-positive precursor cells display a radial distribution. (B^1^-D^3^) After fusion of the neuroepithelium, Pax6/VIM+ cells are arranged mainly as rosette-like structures. (E^1^-E^3^) In the adult pineal gland (PG) individual cells positive for Pax6 and/or vimentin are dispersed throughout the parenchyma. White arrowheads: Pax6^high^/VIM^high^ cells. Yellow arrowheads: Pax6^high^/VIM^low^ cells. White arrows: Pax6^low^/VIM^high^ cells. (A^1^-E^3^) 3x, 4x, 3x, 2.5x and 2.6x digital zooms of the insets shown in Figs 1B^2^–1B^4^, 1F^2^–1F^4^ and 1G^2^–1G^4^ and 2A^2^–2A^4^ and 2C^2^–2C^4^, respectively; scale bar: 10 μm. E, embryonic day. P, postnatal day.

We examined factors that contribute to pineal gland organogenesis and homeostasis, with a special focus on microglial cells. To this end, we first characterized the main stages of pineal gland development. We found that Pax6, a homeobox transcription factor essential for pineal gland formation and pinealocyte specification [12,13,15], and the intermediate filament protein vimentin (VIM), both strongly labeled neuroepithelial cells in the pineal primordium (Fig 1). Pax6/VIM double immunostaining highlighted the pinealocyte precursor cell lineage and the dynamics of cellular arrangements throughout pineal gland ontogeny. The expression of Pax6 mRNA in the developing pineal gland has been well defined, with the highest levels recorded during embryogenesis and decreasing mRNA expression during the postnatal period [17]. Here we show for the first time, to the best of our knowledge, a detailed ontogenetic characterization of the cells that express Pax6 protein in the developing and mature pineal gland, using immunohistochemistry (Figs 1–3).

The neuroepithelial precursor cells in the forming pineal gland expressed both Pax6 and vimentin, as in the developing cerebral cortex [37]. The pinealocyte precursor cells also displayed other similarities with cortical precursor cells. For example, during early phases of tubular elongation of the pineal gland, at E15 and E16 in the rat, the Pax6/VIM double-positive cells were arranged in a radial fashion (Figs 1A^1^–1B^4^ and 3A^1^–3A^3^), as in the neuroepithelium of the dorsal cortex. By E17, the pineal neuroepithelium begins to fold and fuses at the midline (Fig 1C^1^–1C^4^). By E18, rosette-like structures, comprising Pax6/VIM+ cells, became apparent in the developing rat pineal gland (Figs 1D^1^–1G^4^ and 3B^1^–3B^3^). Rosette-like configurations have also been reported in other species, such as birds, and thus appear to be a common feature of pineal growth [38]. The number of rosettes decreased during the first two weeks after birth, and by that time the Pax6/VIM+ cells in the gland exhibited a more homogeneous distribution (Figs 2A^1^–2B^4^ and 3D^1^–3D^3^).

**Fig 4:**
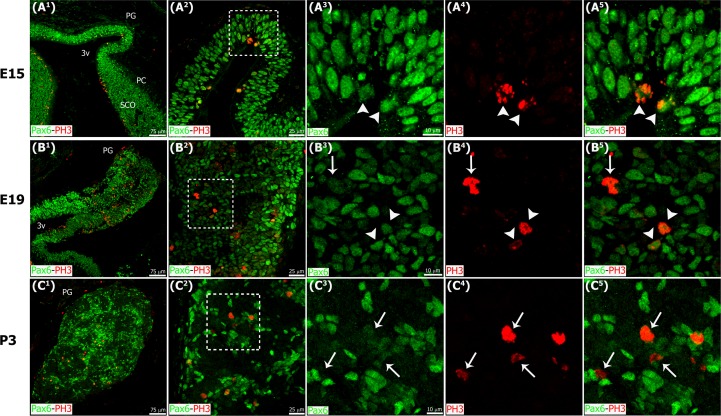
Actively dividing precursor cells express Pax6, and they are located at the surface of the ventricular lumen of the developing pineal gland at early stages of development, and dispersed throughout the gland after fusion of the neuroepithelium. Confocal microscopy of sagittal sections through the rat pineal gland (PG) immunolabeled for Pax6 (green) and the mitotic M-phase marker phospho-histone H3 (PH3, red). All PH3+ mitotic cells expressed Pax6; Pax6 levels, however, varied among dividing pinealocyte precursor cells. The number of mitotic precursor cells increased progressively during embryogenesis, and dropped quickly after birth. White arrowheads: Pax6^high^ mitoses. White arrows: Pax6^low^ mitoses. (A^1^-C^1^) 20x; scale bar: 75 μm. (A^2^-C^2^) 60x; scale bar: 25 μm. (A^3^-C^5^) 3x digital zooms of the insets shown at 60x; scale bar: 10 μm. E, embryonic day. PC, posterior commissure. P, postnatal day. SCO, subcommissural organ. 3v, third ventricle.

We unequivocally identified the precursor cells of the developing pineal gland by labeling actively dividing cells with the mitotic M-phase cell marker phospho-histone H3 (PH3) [39]. At E15, all PH3+ mitotic cells in the gland were located along the ventricular surface and expressed Pax6 (Fig 4A^1^–4A^5^). The location of Pax6+ mitotic cells in the E15 and E16 pineal gland matched the position of Pax6+ precursor cells at early stages of development in the cerebral cortex [37]. However, by E18 or E19, after the pineal neuroepithelium had fused, the PH3+ mitoses were more abundant and evenly distributed throughout the gland (Fig 4B^1^–4B^5^). The random distribution of mitotic cells was maintained in the perinatal pineal gland (Fig 4C^1^–4C^5^). Nonetheless, at each stage of development all PH3+ dividing cells expressed detectable levels of Pax6 regardless of location. We did not observe PH3+ mitoses in the adult pineal gland.

We noted that Pax6 and vimentin expression levels varied according to the stage of development. In general, Pax6 and vimentin expression was strongest at the earliest phases of pineal gland formation and decreased through pre- and postnatal development. Early in pineal ontogeny, most cells were Pax6/VIM double positive (Figs 1 and 3A^1^–3B^3^), but as development proceeded some individual cells that expressed mainly Pax6 or vimentin were also present and randomly distributed throughout the gland (Figs 2 and 3C^1^–3E^3^). Pax6^high^/VIM^high^, Pax6^high^/VIM^low^, and Pax6^low^/VIM^high^ cells were clearly distinguishable at these later ages, and some strongly expressing Pax6+ cells remained in the adult gland (Fig 3C^1^–3E^3^). Among the PH3+ mitotic cells we observed variable Pax6 levels, but all dividing cells expressed this essential homeobox transcription factor. We noted that the level of Pax6 expression in dividing cells decreased over the course of pineal organogenesis, since the proportion of actively dividing cells that were Pax6^low^ increased (Fig 4). Previous BrdU birth dating studies in rat have shown that the vast majority of pineal cells are generated between E18 and P5 [40,41]. Our data show that all dividing cells expressed Pax6 during this same developmental window, indicating that Pax6+ precursor cells produce the pinealocyte lineage. We also noted that Pax6-expressing cells were still present in the adult gland, distributed evenly across the organ (Figs 2C^1^–2C^4^ and 3E^1^–3E^3^) [42]. Many of the Pax6+ cells in the adult pineal gland also expressed detectable levels of vimentin (Fig 3E^1^–3E^3^), suggesting the possibility that they may also generate astroglial cells.

**Fig 5:**
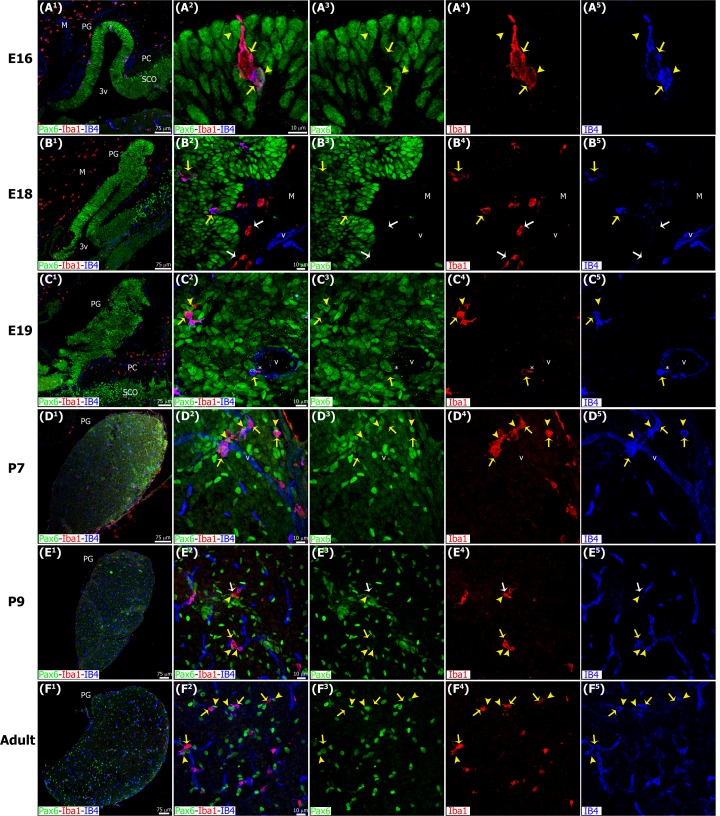
Microglia colonize the pineal gland neuroepithelium very early in development and persist across the lifespan, showing affiliation with Pax6+ cells. Microglia in the developing and adult pineal gland (PG) exhibit an ‘activated’ morphology. Panels show images of confocal microscopy of PG sections immunolabeled for Pax6 (green) and the microglial marker Iba1 (red), and stained with the isolectin IB4 (IB4, blue) to reveal blood vessels (v) and a subpopulation of microglial cells. Iba1+ microglia were present in the meninges (M) and choroid plexus, and appeared to enter the PG from both sources early in development. In a few cases microglial cells were seen phagocytosing Pax6+ precursor cells (yellow arrowheads) in early postnatal development (A^1^-A^5^), but this was most common in the postnatal and adult PG. Yellow arrows show Iba1/IB4 double-positive cells. The tight spatial relationship between macrophages and blood vessels suggests that microglia might also colonize the PG through the vasculature (asterisk). White arrows: microglial cells positive for Iba1 and negative for IB4 (red). (A^1^, B^1^) 20x; (C^1^, E^1^) 1.6x and 1.2x digital zooms from 10x images, respectively; (D^1^ and F^1^) 10x; scale bar: 75 μm. (A^2^-A^5^, B^2^-B^5^, C^2^-C^5^, D^2^-D^5^, E^2^-E^5^) 3x, 1.4x, 1.7x, 1.5x and 1.4x digital zooms from 60x images, respectively; (F^2^-F^5^) 1.5x enlargements from a 40x image; scale bar: 10μm. E, embryonic day. M, meninges. P, postnatal day. PC, posterior commissure. SCO, subcommissural organ. 3v, third ventricle.

**Fig 6:**
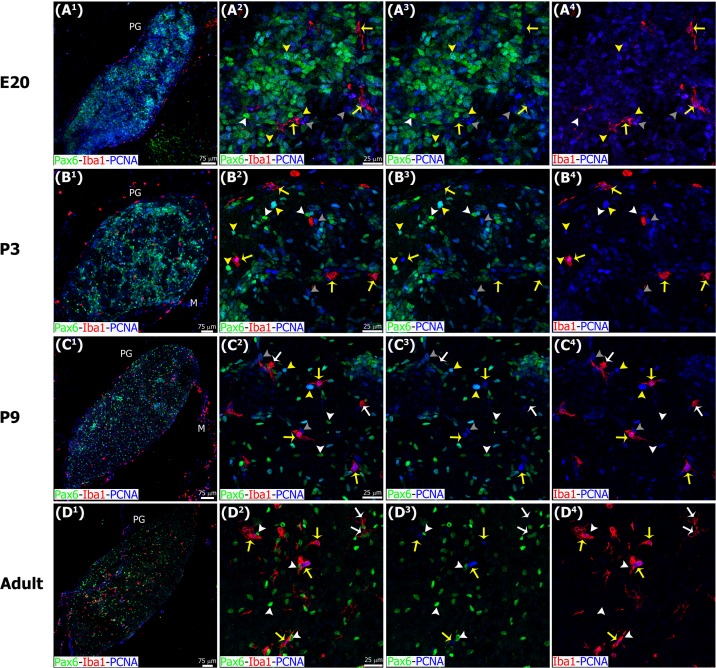
Microglia express the mitotic cell marker PCNA throughout the entire pineal gland ontogeny, while Pax6+ cells have detectable PCNA levels mainly during early development. Panels show images of confocal microscopy of pineal gland (PG) sections immunostained for PCNA (blue), Pax6 (green) and Iba1 (red). The combination of these three markers revealed cell heterogeneity in the pineal gland in the different stages analyzed. Yellow arrows: Iba1/PCNA double-positive microglial cells. White arrows: microglia positive for Iba1 and negative for PCNA. Yellow arrowheads: Pax6/PCNA double-immunoreactive cells. White arrowheads: Pax6+ precursor cells negative for PCNA. Grey arrowheads: cells positive only for PCNA. (A^1^, C^1^, D^1^) 1.5x, 1.3x and 1.1x digital zooms from 10x images, respectively; (B^1^) 20x; scale bar: 75 μm. (A^2^-C^4^) 60x; (D^2^-D^4^) 1.4x digital zooms from a 40x image; scale bar: 25 μm. E, embryonic day. M, meninges. P, postnatal day.

In addition to Pax6/VIM+ precursor cells in the pineal primordium, we also identified Iba1-expressing microglial cells, which are the resident macrophages of the central nervous system (CNS). We found that microglia begin to colonize the pineal neuroepithelium by E15, and were present at all stages of pineal gland development (Fig 5). The meninges surrounding the gland, and the choroid plexus of the third ventricle appeared to be two sources of colonizing microglial cells. The population of microglial cells in the pineal gland increased across the span of development, achieving the density of cells present in the adult gland during the second postnatal week. Many of the pineal microglia stained positive for the mitotic cell marker PCNA, indicating that they retained the capacity to divide (Fig 6). This suggests that *in situ* proliferation may contribute to the increased number of microglia that we observed during pineal development. At the adult stage, the Iba1+ microglia exhibited a nearly even distribution throughout the gland (Fig 6D^1^–6D^4^).

The morphology of Iba1+ microglia in the developing pineal gland corresponded to that of ‘activated’ cells. The microglia had prominent round or oval somas and a few short thick processes that labeled strongly for Iba1. Some of the microglia had an amoeboid shape. The morphology of pineal microglia matched that of microglia in the developing rat cerebral cortex [39,43]. Combined staining with the conjugated isolectin IB4 revealed at least two subpopulations of microglial cells. Most microglia in the gland stained positive for both Iba1 and IB4. However, microglia located in the meninges surrounding the pineal gland mostly stained only for Iba1 (Fig 5). Interestingly, we noted that the Iba1+ microglial cells in the adult pineal gland maintained the appearance of activated cells (Figs 5F^1^–5F^5^ and 6D^1^–6D^4^). This stands in marked contrast to the morphology of microglial cells in other healthy adult CNS structures, such as the neocortex, where microglia exhibit a ‘resting’ morphology characterized by a relatively small soma with many fine ramified processes.

**Fig 7:**
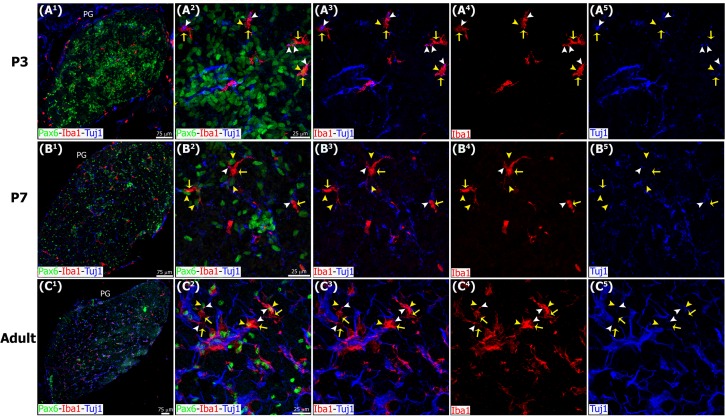
Microglia interact with Tuj1+ nerve fibers in the postnatal and adult pineal gland. Sections of postnatal (P) and adult pineal glands (PG) immunolabeled for Pax6 (green), Iba1 (red) and β-tubulin III (Tuj1, blue). Sympathetic nerve fibers and microglia form an intricate network where Pax6+ cells are located. Macrophages (yellow arrows) phagocytosing Pax6+ cells (yellow arrowheads) and/or pruning nerve fibers (white arrowheads) are indicated. (A^1^-B^1^) 20x; (C^1^) 10x; scale bar: 75 μm. (A^2^-A^5^, B^2^-B^5^, C^2^-C^5^) 1.3x, 1.4x and 1.2x digital zooms from 60x images, respectively; scale bar: 25 μm.

**Fig 8:**
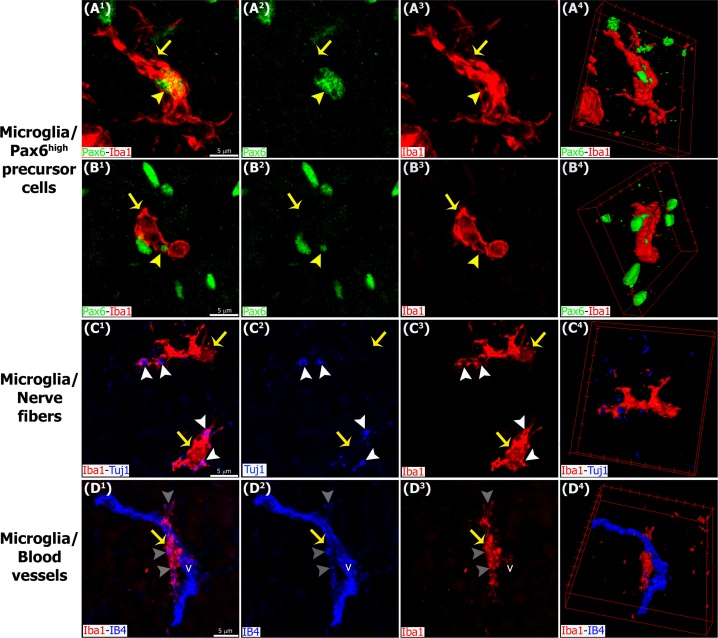
Higher magnification images demonstrating interactions between microglia, Pax6+ cells, nerve fibers and blood vessels in the pineal gland. Panels show confocal images of pineal gland (PG) immunolabeled for Pax6 (green), Iba1 (red) and β-tubulin III (Tuj1, blue), or IB4-stained blood vessels (v, blue). Microglia (yellow arrows) closely affiliate with and in some cases engulf Pax6+ precursor cells (yellow arrowheads) (A^1^-B^4^), Tuj1+ nerve fibers (white arrowheads) (C^1^-C^4^) and blood vessels (grey arrowheads) (D^1^-D^4^) within the PG. (A^1^-B^3^, C^1^-C^3^, D^1^-D^3^) 4x, 3.4x and 3.5x digital zooms from 60x images, respectively; scale bar: 5 μm. (A^4^, B^4^, C^4^, D^4^) 3D reconstruction of the main interactions.

**Fig 9:**
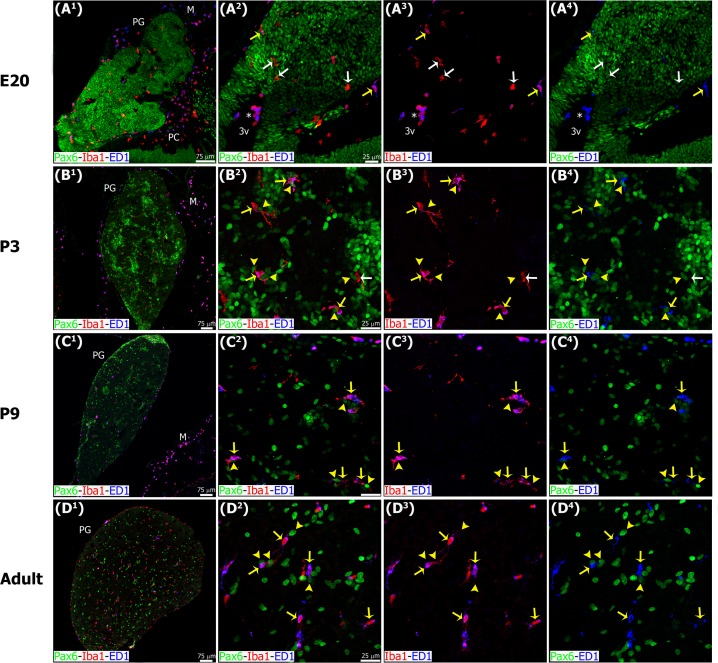
Microglia phagocytic potential increases throughout pineal ontogeny. Panels show confocal microscopy of sections immunostained for the lysosomal marker ED1 (CD68, blue), Pax6 (green) and Iba1 (red). (A^1^-B^4^) Late embryonic (E20) and early postnatal (P3) pineal gland (PG) sections showing two subpopulations of microglial cells with (yellow arrows) or without (white arrows) ED1 expression. (C^1^-D^4^) Later neonatal (P9) and adult PG exhibiting the majority of Iba1/ED1 double-immunoreactive cells in close proximity to or phagocytosing Pax6^high^ cells (yellow arrowheads). Asterisk, cluster of round Iba1/ED1 double-positive cells in the lumen of the third ventricle (3v) and in close proximity to the proximal pineal gland. (A^1^) 20x; (B^1^) (C^1^, D^1^) 1.2 x and 1.3x digital zooms from 10x images, respectively; scale bar: 75 μm. (A^2^-A^4^) 40x; (B^2^-D^4^) 60x; scale bar: 25 μm. M, meninges. PC, posterior commissure.

**Fig 10:**
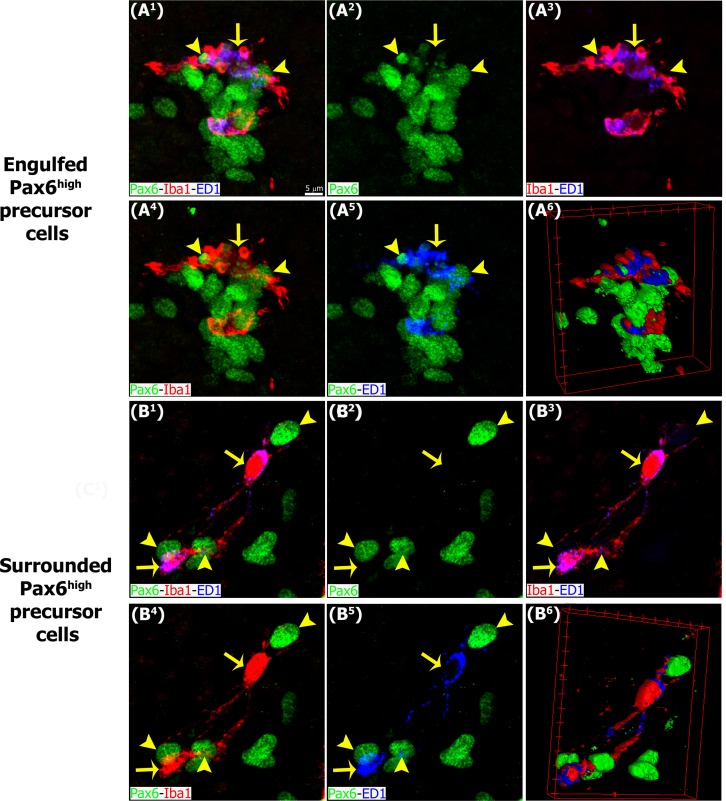
Activated microglia contact and engulf Pax6+ precursor cells. Higher magnification of pineal gland sections immunolabeled for Iba1 (red, yellow arrows), ED1 (blue, yellow arrows) and Pax6 (green, yellow arrowheads). (A^1^-A^6^) Microglial cells with an activated morphology are enriched in cytoplasmic ED1-positive bodies and engulf Pax6^high^ nuclei and elements. (B^1^-B^6^) Microglia with few thick projections contacting and surrounding Pax6^high^ precursor cells. (A^1^-A^5^, B^1^-B^5^) 4x and 3.6x digital zooms from 60x images, respectively; scale bar: 5 μm. (A^6^, B^6^) 3D reconstruction of phagocytic microglia/Pax6^high^ precursor cells or elements interactions.

**Fig 11:**
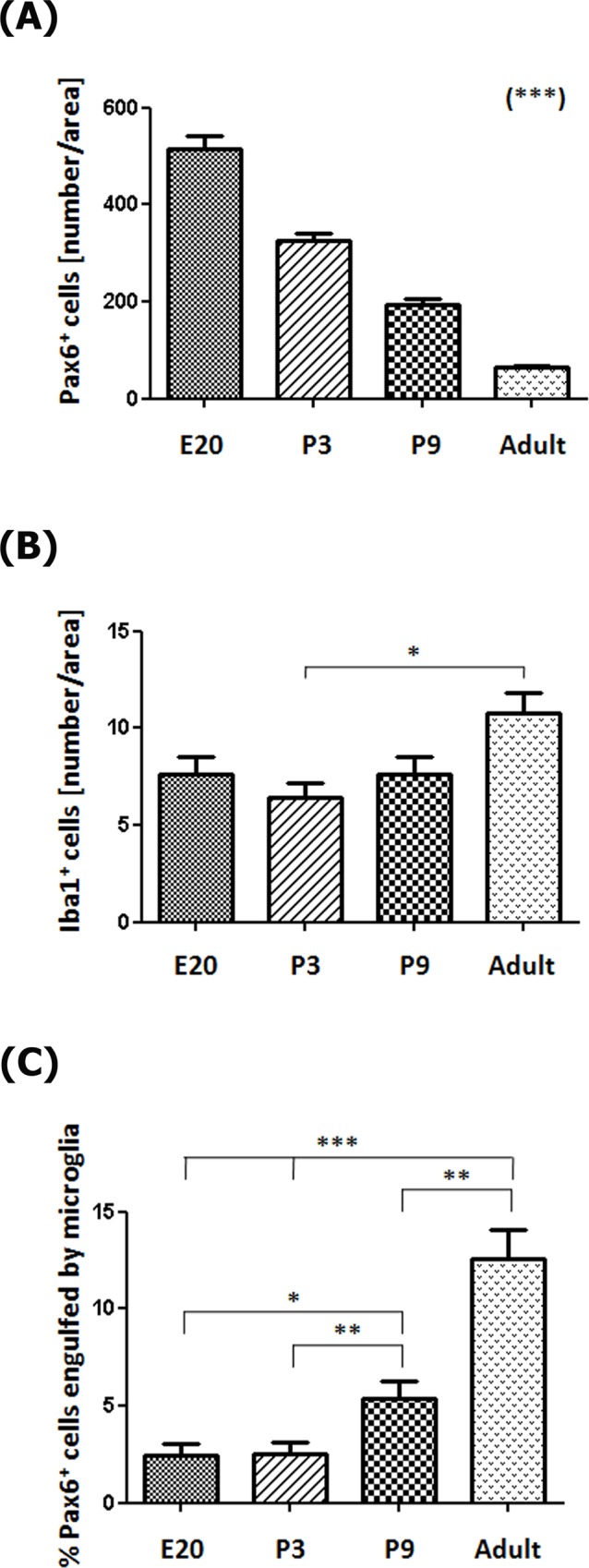
Morphometric analysis shows that Pax6+ cell numbers decrease, while Iba1+ cell numbers, and Pax6+ cells engulfed by microglia increase in the pineal gland through ontogeny. (A) The number of Pax6+ precursor cells per area (0.05 mm^2^) decreases throughout pineal development (Post-test for linear trend: *P* < 0.0001). (B) Microglia density increases slightly between postnatal day 3 (P3) and adulthood (Post-test for linear trend: *P* < 0.05). (C) The percentage of Pax6+ precursor cells in contact or engulfed by microglial cells is higher in late neonatal stages onwards (Post-test for linear trend: *P* < 0.0001). Data were expressed as mean ± S.E.M. Statistics: one-way ANOVA followed by the Tukey post-test: *** *P* < 0.001, ** *P* < 0.01, * *P* < 0.05. E, embryonic day.

During early pineal development, microglia were seen in close proximity to Pax6+ cells in the neuroepithelium, but we noted very few examples of phagocytosis (Fig 5A^1^–5A^5^), as shown in the developing neocortex [39]. However, microglial phagocytosis of Pax6+ cells increased slowly over development, and was quite common in the adult pineal gland. Figs 5, 6, 7, 8A^1^–8B^4^, 9 and 10, S1 and S2 Figs, and S1 and S4 Videos show examples of Pax6+ cells surrounded, enveloped, or phagocytosed by microglia. Furthermore, co-immunostaining with the lysosomal marker ED1 (CD68), a marker of active phagocytosis, revealed a gradient of increased phagocytic capacity in the pineal gland across development and into adulthood (Figs 9 and 10, S2 Fig, and S4 Video). We performed a morphometric analysis of the ontogeny of Pax6+ cells, Iba1+ microglia, and their interactions. These data revealed decreasing expression of the Pax6 transcription factor, a significant increase of activated microglial cells in the adult pineal gland, and a concomitant enhancement of microglial interconnections with, and engulfment of, Pax6+ cells (Fig 11).

**Fig 12:**
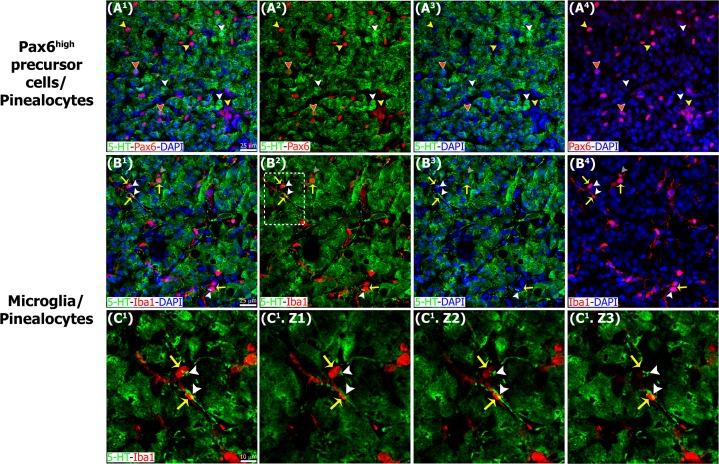
Pinealocytes appear to be protected from microglia-mediated phagocytosis. Sections of adult pineal gland immunolabeled for serotonin or 5-hydroxytryptamine (5-HT, green), Pax6 (red) and Iba1 (red). DAPI (blue) was used as general nuclear dye. (A^1^-A^4^) Pinealocytes positive for 5–HT (white arrowheads) and Pax6+ cells (yellow arrowheads) represent two distinct cell populations. Few cells positive for both Pax6 and 5-HT were identified (orange arrowheads with white borders). (B^1^-B^4^) Microglia (yellow arrows) in close proximity to 5-HT-immunolabeled projections (white arrowheads) and phagocytosing a serotonin-negative cell (grey arrowheads). (C^1^) Higher magnification of the inset shown in B^2^. (C^1^.Z1-C^1^.Z3) Three consecutive confocal planes (Z) that show the points of contact between microglia and serotonergic processes. (A^1^-B^4^) 1.4x digital zooms from 40x images; scale bar: 25 μm. (C^1^-C^1^.Z3) 3.2x digital zooms from the inset at 40x shown in B2; scale bar: 10 μm.

We also examined interactions between microglial cells and other constituents of the developing and mature pineal gland. Pinealocytes comprise approximately 95% of all cells in the adult pineal gland and can be identified by expression of serotonin (5-hydroxytryptamine; 5-HT), a precursor of the hormone melatonin. We noted that most of the 5-HT+ pinealocytes did not express Pax6. Pinealocytes and Pax6+ cells were clearly distinguishable by nuclear morphology and location within the gland. Pinealocytes formed follicle- and cord-like structures, while Pax6+ cells were isolated or located in small clusters adjacent to vascular elements (Fig 12A^1^–12A^4^). We found that the 5-HT+ pinealocytes were rarely engulfed by microglial cells. However, microglial interactions with pinealocyte processes were observed (Fig 12B^1^ and 12C^1^.Z3). We noted intricate networks of closely apposed microglial cells, Pax6+ cells, nerve fibers and blood vessels (Figs 5 and 7). We observed that microglial cells also phagocytosed nerve fibers stained with the Tuj1 neuronal marker, and elements of the vasculature labeled with IB4 (Fig 8C^1^ and 8D^4^, S1 Fig, and S2 and S3 Videos).

Together, our data demonstrate that Pax6-expressing cells in the pineal gland serve as the precursor cell for pinealocytes and some interstitial cells, and that microglia actively contribute to pineal gland organogenesis and homeostasis through phagocytosis of precursor cells, and remodeling of blood vessels and nerve fibers.

## Discussion

The pineal gland is composed mainly of pinealocytes that release melatonin, astrocytes, microglia, endothelial cells, and, in some species, a small number of neurons [18]. Based on their nuclear characteristics, adult rat pinealocytes have been classified into two types by Calvo and Boya [44]. Type I pinealocytes (85–90% of parenchymal cells) present large, round euchromatic nuclei with prominent nucleoli, while Type II pinealocytes were described as less abundant (10–15% of cells) with small, oval nuclei that appear dense at the light microscopy level. Their lineages and specific functions, to the best of our knowledge, have not been unraveled. Pineal developmental dynamics need to be further studied although the functioning of the adult pineal gland as the main melatonin source is well understood. Here we used immunostaining and confocal microscopy to identify the cytoarchitectural features that contribute to pineal gland formation.

It has been shown that Pax6 gene expression is essential for pineal gland formation [12,13,15], and that Pax6 mRNA level in the pineal gland peaks during embryonic development [17]. However, the specific cells that express Pax6 protein in the pineal gland have not been identified, nor characterized in terms of their location in the developing gland, their function, or their relationship and interactions with other cells in the pineal gland. Determining these features will increase our understanding of factors that regulate pineal gland formation. We describe here cell types that are present in the rat pineal gland from the earliest stages of development through adulthood. Our data show that the pineal recess develops from a population of Pax6-expresssing neuroepithelial cells (Fig 1), and that all mitotic cells in the developing and early postnatal pineal gland are positive for this homeobox transcription factor. The Pax6+ cells initially underwent division at the surface of the ventricle in the pineal primordium (Fig 4A^1^–4A^5^), as occurs in the cerebral cortex [45]. After the pineal neuroepithelium had fused, the Pax6+ mitoses were distributed evenly throughout the gland (Fig 4B^1^ and 4C^5^). Pax6+ expression at the cellular level has been detailed in developing CNS structures, such as the cerebral cortex [46]. Pax6+ precursor cells have been shown to produce dedicated neuronal progenitor cells, excitatory glutamatergic cortical neurons, astrocytes, and oligodendrocytes in the developing dorsal telencephalon in a variety of mammalian species [37]. The data we present here show that all dividing cells express Pax6 during the proliferative window of pineal development. This strongly suggests that Pax6+ precursor cells are the progenitor of 5-HT^+^ pinealocytes (Fig 12) and pineal astroglial cells. The multipotency of pineal Pax6-immunoreactive cells is currently being investigated.

Interestingly, we found that a small population of Pax6+ cells remained dispersed throughout the adult pineal gland (Figs 2C^1^–2C^4^ and 3E^1^–3E^3^). Most of these cells were negative for the mitotic marker PCNA (Fig 6D^1^–6D^4^) and their nuclear morphology resembles that of the Type II pinealocytes, as described by Calvo and Boya [44]. Pax6+ cells in the adult pineal gland appear to represent a distinctive cell type that is mainly found in perivascular locations (Fig 12A^1^–12A^4^). In the mature gland, a few examples of Pax6/5-HT double-immunolabeled cells were seen, which indicates that the Pax6+ cells may give rise to or differentiate into pinealocytes, as needed under specific stimuli. However, mitotic M-phase cells are exceedingly rare in the normal adult pineal gland [40,41,47], which suggests the possibility that, in adulthood, pineal Pax6+ cells are quiescent precursor cells. Indeed, birth dating studies provide evidence that precursor cells in the developed pineal gland are capable of synthesizing DNA, but under normal circumstances the precursor cells do not undergo mitosis [47]. Our data show that the majority of Pax6+ cells in the adult pineal gland express the intermediate filament vimentin (Fig 3E^1^–3E^3^), which is consistent with a precursor cell phenotype, and may also suggest a glial cell phenotype. Expression of the two key markers Pax6 and vimentin highlight cytoarchitectural dynamics during pineal gland formation, from an initial radially aligned distribution of precursor cells that transition into rosette-like formations and finally dispersed cells (Figs 1–3).

Microglia have been identified as constituent cells of the pineal gland, based on expression of markers including OX6 (MHCII), OX42 (CD11b), IL-1β, ED1 (CD68), and TNF-R1 [20–26]. We show for the first time that microglia begin to colonize the rat pineal neuroepithelium at the earliest stage of gland formation before pinealocyte differentiation has commenced (Fig 5A^1^–5A^5^). This timing of microglial colonization is a common feature in the developing brain [48]. Microglia appear to populate the pineal primordium from the meninges and also from the choroid plexus of the third ventricle. By adulthood microglia in the pineal gland display an even distribution, as in the cerebral cortex (Figs 5F^1^–5F^5^ and 6D^1^–6D^4^) [39]. Most microglia in the pineal gland express the mitotic marker PCNA (Fig 6), which suggests that *in situ* proliferation may be a third source of microglial cells.

Our data show that microglia in the developing pineal gland exhibited an ‘activated’ morphology. This has been noted in other developing CNS structures, such as the cerebral cortex [39]. Why microglia in many regions of the developing brain display the activated phenotype has not yet been determined, but may be related to the developmental functions attributed to microglial cells, including regulation of cell number and spatial patterning of CNS cells, myelination, and formation and refinement of neuronal circuits [48].

After development is complete, the morphology of microglia in many regions of the brain transitions, and the cells exhibit a ‘resting’ morphology. For example, microglia in the mature healthy cerebral cortex have a relatively small soma and many finely branched processes. Only under pathological conditions microglia in the mature cerebral cortex revert to an activated state. After stroke, injury, infection, cell death or damage in the adult brain, microglia acquire the activated morphology associated with migration and proliferation, phagocytosis of cellular debris, and production of neurotoxic and neurotrophic factors [49,50]. Interestingly, we found that microglia retained an activated mophology in the healthy adult pineal gland. The maintenance of this microglial phenotype may be associated with the special status of the pineal gland as an organ that is not isolated from the body by the blood brain barrier [51]. Microglia appear to be capable of modulating both the activation and down-regulation of the adaptive immune responses in the CNS. Our data showing persistent activation of microglia, and the identification of a cluster of genes related to immune/inflammatory responses differentially expressed in the adult pineal gland [52], suggest a potential immune-related functional pineal specialization within the CNS that requires further attention and examination.

In addition to the differential morphologies, we noted important differences in the behavior of microglia in the developing and mature pineal gland, compared to that reported in other CNS structures. Microglia in the developing mammalian cerebral cortex have been shown to phagocytose Pax6+ and Tbr2+ neural precursor cells, especially in primates [39]. In contrast, we found very few examples of Pax6+ precursor cell phagocytosis in the prenatal pineal gland (Figs 5, 6 and 11). However, we noted that microglial phagocytosis of Pax6+ cells occurred in the postnatal gland, and was more common in the adult pineal gland (Figs 5, 6, 7, 8A^1^–8B^4^, 9, 10 and 11 and S1 and S2 Figs, and S1 and S4 Videos). The clear difference in phagocytic activity in the developing versus adult pineal gland, and in morphology in the adult pineal gland versus adult cerebral cortex demonstrates strong temporal and regional regulation of microglial cell functions. Grabert et al. [53] applied genome-wide analysis of microglia from discrete mouse brain regions, and they found that microglia transcriptional identities and aging susceptibility vary from one location to another. This suggests a differential behavior of microglial cells in neurodegenerative processes involving neuroinflammatory mechanisms. Iba1 immunolabeling by itself did not allow us to distinguish microglial subtypes within the rat pineal gland. However, we observed evidence of microglial diversity by combining Iba1 with the isolectin IB4, and with the lysosomal marker ED1 (Figs 5 and 9). Heterogeneous topographic distribution of microglia in the adult pineal gland immunolabeled for OX42, OX6, ED1 and ED2, as reported by Jiang-Shieh et al. [25], suggests that the prevalence of a particular microglial phenotype might be influenced by current environmental conditions or demands.

Within the pineal gland, microglia have been recognized as part of an intricate cellular network that modulates not only melatonin production but also innate immune responses triggered by invasive and non-invasive stimuli. Functionally, pineal microglia have been reported to serve as: 1) cytokine-dependent mediators of pinealocyte neurite extension and *in vitro* neurotrophic effects [27–30]; 2) antigen-presenting cells via MHC class II (OX6), and blood-derived substances selectors [20,22]; 3) sensors of injury following a non-penetrative blast, intravenous injections of proinflamatory bacterial wall components, or hypoxic exposure [21,31,32]; and 4) regulators and targets of pineal melatonin [31–35]. Together, these studies support the concept that pineal microglia mediate innate immune responses within the CNS by altering melatonin levels via dynamic interactions with astrocytes and pinealocytes. This function is facilitated by pineal microglia exposure to signaling molecules and environmental stimuli that originate outside of the brain. Our data expand the functional role of microglia in the pineal gland by showing that they modulate growth and homeostasis through the phagocytosis of precursor cells, and may also regulate blood flow and signal transduction in the adult gland via phagocytosis of blood vessels and nerve fibers (Fig 8C^1^–8D^4^ and S1 Fig, and S2 and S3 Videos).

## Conclusions

In summary out data are consistent with the concept that Pax6+ cells in the developing pineal gland serve as the precursor cell for the majority of cell types that are found in the adult pineal gland, with the exception of microglial cells. Surprisingly, we find that Pax6+ cells remain in the adult gland, but their functional role remains to be fully understood. Our data also show that microglial cells play a prominent role in the developing gland, and also in the adult gland. Future studies should examine whether and how microglial cell function changes in response to light and dark phases. Finally, our data highlight the intricate affiliation between microglial cells, Pax6-expressing cells, nerve fibers and blood vessels in the mature gland, indicating that these interactions are integral for normal pineal function.

## Supporting Information

S1 Fig Microglial cell interactions with Pax6+ precursor cells, nerve fibers and blood vessels within the pineal gland captured in consecutive confocal planes. Immunolabeling for Pax6 (green), Iba1 (red) and β-tubulin III (Tuj1, blue). Blood vessels (v) were revealed with isolectin IB4 conjugated with a fluorophore (blue). Confocal images from five successive optical sections (Z) are displayed. These confocal planes were used to generate the images shown in Fig 8. Elements positive for Pax6 (yellow arrowheads), Tuj1 (white arrowheads) and IB4 (grey arrowheads), markers of precursor cells, nerve fibers and blood vessels, respectively, are seen internalized in the microglia somas and projections (yellow arrows). (A^1^.Z1-B^1^.Z5, C^1^.Z1-C^1^.Z5, D^1^.Z1-D^1^.Z5) 4x, 3.4x and 3.5x digital zooms from 60x images, respectively; scale bar 5 μm.
(TIF)

S2 Fig Interactions between activated microglia and Pax6+ precursor cells captured in sequential confocal planes. These confocal planes (Z) were used to generate the images shown in Fig 10. Immunolabeling for Iba1 (red, yellow arrows), ED1 (blue, yellow arrows) and Pax6 (green, yellow arrowheads). Pax6^high^ elements engulfed and Pax6^high^ cells completely surrounded by microglial cells and their few thick projections are seen. (A^1^.Z1-A^1^.Z5, B^1^.Z1-B^1^.Z5) 4x and 3.6x digital zooms from 60x images, respectively; scale bar: 5 μm.
(TIF)

S1 Video 3-dimensional (3D) representation of Pax6+ elements phagocytosed by an Iba1+ microglial cell shown in Fig 8B^4^. (MP4)

S2 Video 3D representation of Tuj1+ elements phagocytosed by Iba1+ microglia shown in Fig 8C^4^. (MP4)

S3 Video 3D representation of IB4+ blood vessel/Iba1+ microglial cell interactions shown in Fig 8D^4^. (MP4)

S4 Video 3D representation of Pax6+ cells and Iba1/ED1+ microglia interactions shown in Fig 10B^6^. (MP4)
