# Supplementary figures and images for: Cellular Basis of Pineal Gland Development: Emerging Role of Microglia as Phenotype Regulator
Source: PLoS One. 2016 Nov 18;11(11):e0167063. doi: 10.1371/journal.pone.0167063 (PMC5115862; doi:10.1371/journal.pone.0167063)

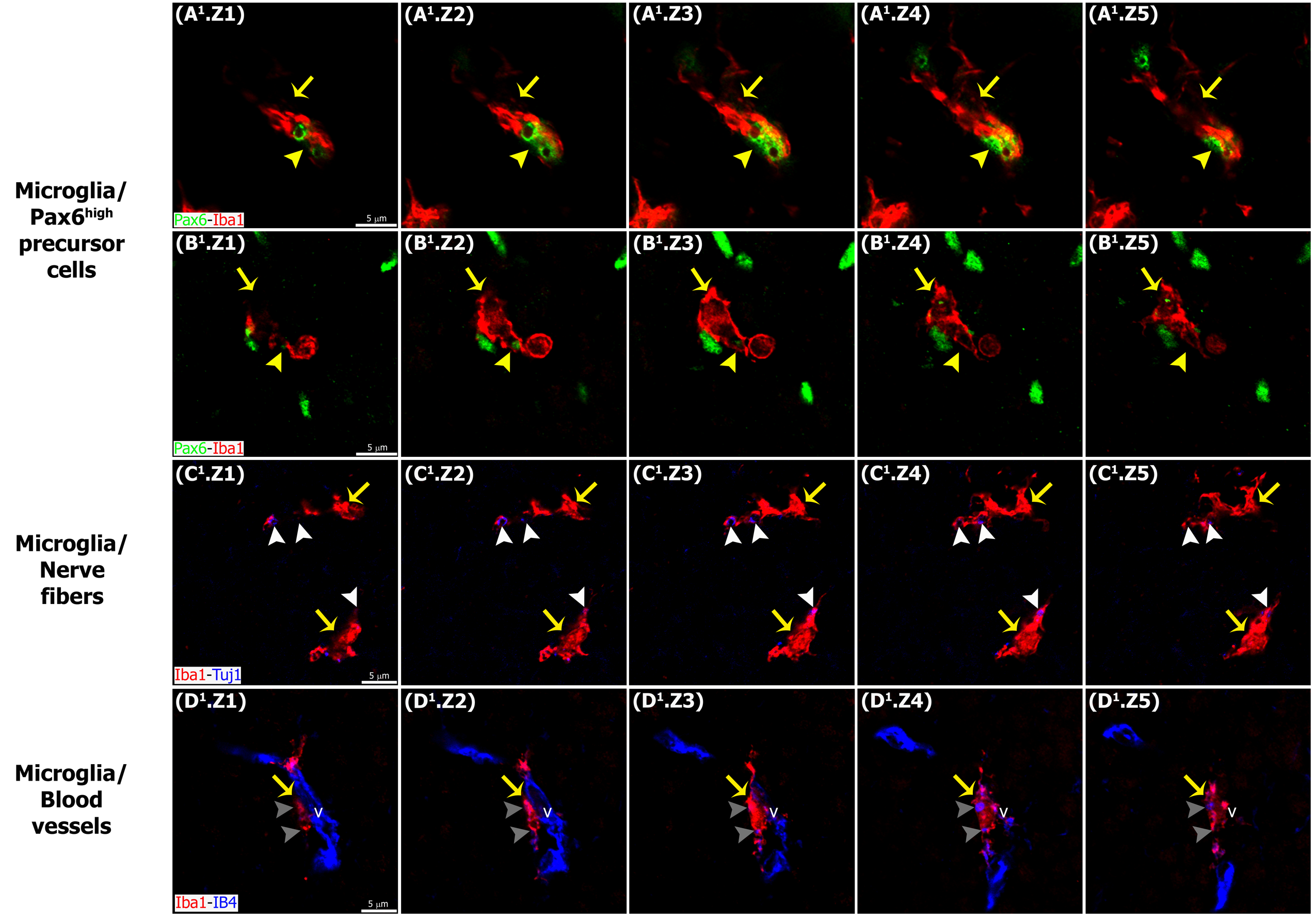

Supplement: S1 Fig — Immunolabeling for Pax6 (green), Iba1 (red) and β-tubulin III (Tuj1, blue). Blood vessels (v) were revealed with isolectin IB4 conjugated with a fluorophore (blue). Confocal images from five successive optical sections (Z) are displayed. These confocal planes were used to generate the images shown in Fig 8. Elements positive for Pax6 (yellow arrowheads), Tuj1 (white arrowheads) and IB4 (grey arrowheads), markers of precursor cells, nerve fibers and blood vessels, respectively, are seen internalized in the microglia somas and projections (yellow arrows). (A1.Z1-B1.Z5, C1.Z1-C1.Z5, D1.Z1-D1.Z5) 4x, 3.4x and 3.5x digital zooms from 60x images, respectively; scale bar 5 μm. (TIF) [file pone.0167063.s001.tif]

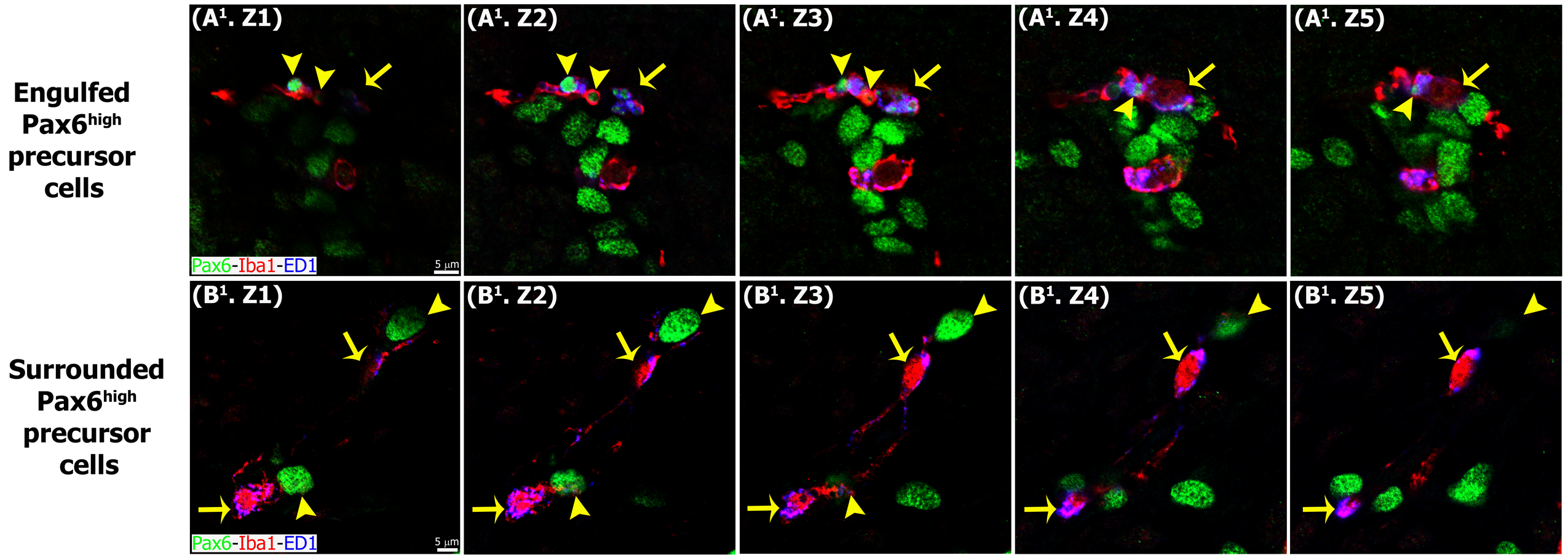

Supplement: S2 Fig — These confocal planes (Z) were used to generate the images shown in Fig 10. Immunolabeling for Iba1 (red, yellow arrows), ED1 (blue, yellow arrows) and Pax6 (green, yellow arrowheads). Pax6high elements engulfed and Pax6high cells completely surrounded by microglial cells and their few thick projections are seen. (A1.Z1-A1.Z5, B1.Z1-B1.Z5) 4x and 3.6x digital zooms from 60x images, respectively; scale bar: 5 μm. (TIF) [file pone.0167063.s002.tif]
